# Supplementary material for: IFN-α/β Signaling Is Required for CDG-Mediated CTL Generation and B Lymphocyte Activation
Source: Pharmaceutics. 2022 Dec 16;14(12):2821. doi: 10.3390/pharmaceutics14122821 (PMC9786310; doi:10.3390/pharmaceutics14122821)
Supplement: Supplementary file 1 [file pharmaceutics-14-02821-s001.zip › pharmaceutics-2084986-supplementary.pdf]

Supplementary Figures:

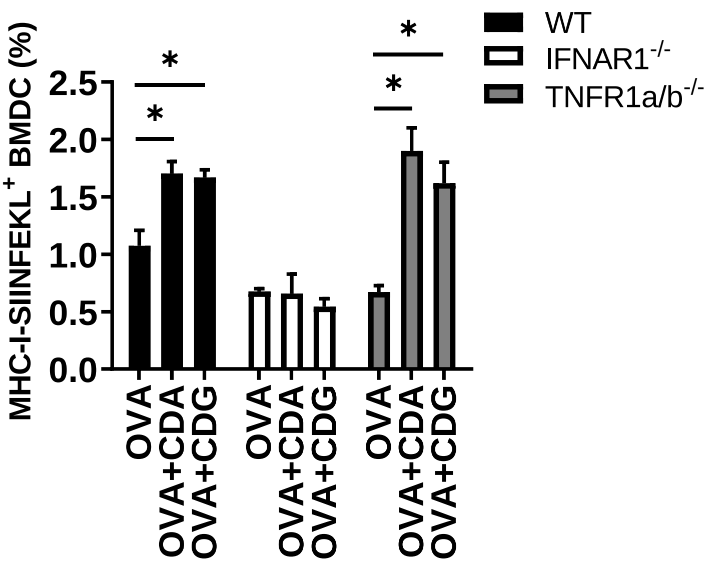

Supplementary Figure S1: Cross-presentation of the OVA immunodominant peptide SIINFEKL in BMDC pulsed with OVA +/- CDN. Cross-presentation of BMDC from IFNAR1-/- and TNFR1a/b-/- mice was compared to WT counterparts in the presence or absence of CDA or CDG. Error bars indicate SEM. Statistical significance was calculated using one-tailed Student's t-test. * indicates p<0.05.

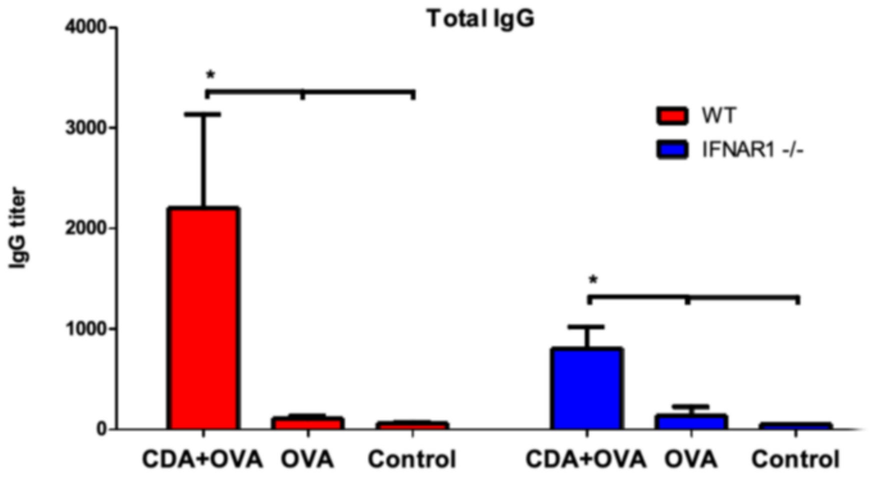

Supplementary Figure S2: Antigen specific IgG titer after vaccination. OVA specific IgG titers from vaccinated WT and IFNAR1$^{-/-}$ mice were determined by ELISA. Error bars indicate SEM. Statistical significance was calculated using one-tailed Student's t-test. * indicates p<0.05. The results are representative of four independent experiments (WT, n=3-4; IFNAR1$^{-/-}$, n=3-4).
